# Quality end-of-life care for dementia: What have family carers told us so far? A narrative synthesis

**DOI:** 10.1177/0269216314526766

**Published:** 2014-07

**Authors:** Nathan Davies, Laura Maio, Greta Rait, Steve Iliffe

**Affiliations:** Research Department of Primary Care and Population Health, University College London, London, UK

**Keywords:** Dementia, quality of health care, palliative care, end-of-life care, caregivers

## Abstract

**Background:**

People with dementia do not always receive good quality end-of-life care, with undertreated pain, aggressive medical interventions and limited access to hospice care being common. Family carers often provide the majority of informal care for people with dementia, therefore may be best placed to comment on quality of care.

**Aim:**

We explored what quality end-of-life care for dementia is from the perspective of family carers.

**Design:**

A review of qualitative evidence taking a systematic approach using a narrative synthesis with tabulation, textual description of studies and thematic analysis as tools, following the guidelines from the Economic and Social Research Council.

**Data sources:**

Keywords and subject headings were searched in MEDLINE, EMBASE, CINAHL, SCIE and PsycINFO for studies from 1990 in April 2012 and updated in May 2013. Reference lists were checked and citation searches undertaken.

**Results:**

Eight studies were included. There was an overarching theme of ‘A family’s belief of death and their choice of treatment’. Three further themes were then identified to explain family carers’ beliefs: the relationship with professionals as a core component of care quality; emotional and commitment pressures of caring and finally, family carers’ ability to think about death and dying.

**Conclusion:**

It is difficult to define what constitutes high-quality end-of-life care for people with dementia from the perspective of family carers. Their views expressed in the literature appear to demonstrate more variation of preference of care and treatment and their uncertainty of this.

**What is already known about the topic?**

- Carers of people with dementia experience high levels of stress, strain and burden.
- People with dementia at the end of life do not always receive high-quality end-of-life care.
- Research of carers of people with dementia has focussed on the diagnosis and transition stages of dementia.

**What this paper adds?**

- Strengthens the call for further exploration of carers’ views about end-of-life care for dementia.
- Carers’ views are mixed and lie on a spectrum of acceptance of their relative as actively dying with dementia.
- Combines the small amount of data available about quality end-of-life care for dementia from the views of carers.

**Implications for practice, theory or policy**

- Greater investment is needed in bereavement research and practice for those who are less accepting of their relative as actively dying both before and after death.
- Carers need to be included in the development of individualised care plans which may replace the fallen Liverpool Care Pathway.
- Professionals should be aware of the differences between carers who have different relationships with the person with dementia such as spouse or adult child, with different priorities and commitments.

## Background

Due to an ageing population worldwide, age-related conditions such as dementia are expected to rise to 115 million worldwide by 2050.^1^ Although the incidence may be falling, the prevalence in the older population may be closer to 6.5% than to the 8.3% sometimes quoted.^2^

The symptoms of end-stage dementia leave individuals unable to care for themselves and dependent on the care of others. The majority of dementia care is initially informal and often provided by relatives or friends of the person with dementia.^3^ These carers are more often spouses, middle-aged adult children or adult children in law of the people with dementia and are more likely to be female,^4–7^ without whom the formal support system would be likely to collapse.^8^ However, as dementia progresses, care is provided more by formal services with the result that the majority of people recorded as having dementia as the underlying cause of death die in care homes and hospitals.^9–11^

Carers have a range of different titles or labels such as lay carers, untrained carers, informal carers or family carers.^12^ Caring can be a stressful occupation, and the burden placed on the individual as a carer may be great, with limited opportunities for breaks, socialising and having what one may classify as a ‘normal’ life. Caring for an older adult or a relative who has dementia is thought to be one of the most stressful and difficult types of caring.^13^

There is a growing body of literature exploring the burden of caring for someone with dementia,^14^ the guilt felt by carers,^15^ pre-death grief^16,17^ and physical and mental health, including stress,^18^ depression,^19^ coping^20,21^ and unmet need,^22^ with most of this surrounding the diagnosis and transition stages of caring. However, more work is also need surrounding the disclosure of diagnosis.^23^ Transition stages describe a change from one stable period/state to another, for example, one of the most significant transitions is the transition from being cared for at home to being cared for in a nursing home. Relatively little is known about their experiences at the end of life.^24^

There are only four reviews which explore the experiences of family carers of people with dementia at the end of life. Ryan^25^ focussed on familial involvement and beliefs surrounding decision-making in end-of-life care (EOLC) for people with dementia. Hennings et al.^26^ focus solely on dying in care homes. Raymond et al.^27^ conducted a rapid appraisal and hence were not systematic in their approach with an aim to focus on both professionals’ and family carers’ personal narratives which were more anecdotal in their approach, as opposed to research articles on carers alone. Finally, Peacock^28^ conducted a broad review of experiences of family carers and end-of-life dementia care but did not have a focus on quality.

It has been previously reported that many people with dementia do not always receive the best EOLC, are often unable to gain access to hospice care,^29^ have undertreated pain and experience avoidable admissions to hospital.^30^ EOLC has recently received much media attention in the United Kingdom which has highlighted areas of poor quality care, in particular hospital care for elderly patients,^31^ EOLC and the use of the Liverpool Care Pathway (LCP).^32^ The LCP was a framework for practitioners to use to guide EOLC; however, following an independent report and much negative media attention from stories by families, the LCP is being phased out by the UK government.^32^

The current review aimed to search and synthesise papers which have explored carer’s experiences, focussing on their experiences and opinions of quality EOLC for dementia. The aim of the study was to explore what is understood about quality EOLC for dementia from the perspective of families?

## Method

### Design

A literature review of qualitative studies was conducted following the Centre for Reviews and Dissemination guidelines taking a systematic approach to the search strategy and selection procedures.^33^ A narrative synthesis was adopted using guidance from the Economic and Social Research Council (ESRC)^34^ with thematic analysis methodology as a tool to present the narrative. Quotations from the original articles included within this review are used within the ‘Results’ section to support the authors’ findings and interpretations in order to enhance the rigour of the analysis.^35^

### Search strategy

Five electronic databases were searched – MEDLINE, EMBASE, CINAHL, SCIE, and PsycINFO – in April 2012, updated in May 2013 and included literature from 1990, with grey literature searched for using System for Information on Grey Literature in Europe (SIGLE).^36^ The year 1990 was selected as the start date for searching literature as this was the start of a movement of research into carers’ needs. Reference lists of included papers and citation tracking were used in PubMed and Google Scholar to identify additional material not found in electronic databases. A search filter, pretested strategy for dementia from the Scottish Intercollegiate Guidelines Network (SIGN),^37^ was used to guide the search for this review. Relevant experts in the field were contacted to identify any additional relevant papers.

A set of keywords was identified and used to scope the literature (see Table 1). From this scoping, synonyms were identified and/or abbreviations that were felt appropriate.

**Table 1.** Search terms used in electronic databases.

	Search terms
Quality of care	‘quality’ or ‘quality of care’
Dementia	‘dementia’ or ‘alzheimer*’ or ‘neurodegenerative*’ or ‘vascular’
End-of-life care	‘palliat*’ or ‘end of life*’ or ‘end of life care’ or ‘eolc’
Family	‘carer’ or ‘family’ or ‘proxy’ or ‘caregiver’ or ‘relative’ or ‘next of kin’ or ‘nok’
Experience	‘perspective’ or ‘perception’ or ‘perceive*’ or ‘view’ or ‘opinion’

Subject headings for keywords were used where possible and adjusted for the different databases; however, not all databases use subject headings for all keywords identified in Table 1. The same keywords were used across all databases.

### Inclusion/exclusion criteria

Papers were included if they met all of the following criteria:

- The clinical focus of the paper was dementia;
- The perspective/view was from that of the family carer of a person with dementia;
- The therapeutic focus was on EOLC or palliative care;
- Perceptions of the quality of care received were reported.

Papers were excluded if they were:

- Not in English language;
- Published prior to 1990;
- Not peer reviewed;
- Quantitative studies.

### Selection procedure

**Figure 1.**
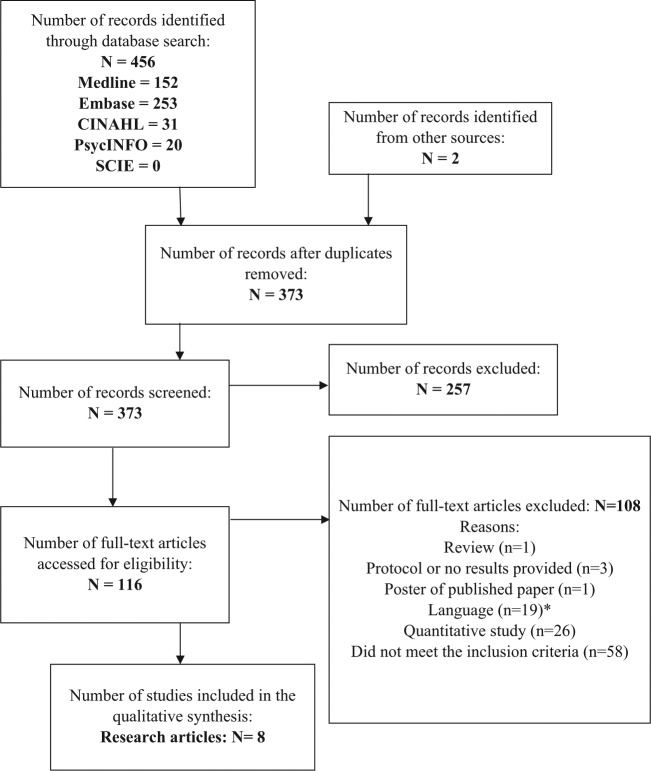
PRISMA flowchart describing the search process of finding articles for quality end-of-life care for dementia from the perspective of family carers.^38^ PRISMA: Preferred Reporting Items for Systematic Reviews and Meta-Analyses. *None of these abstracts met the inclusion criteria.

Titles and abstract were read and assessed for eligibility by one reviewer (N.D.), and the full texts of potentially relevant abstracts, or full papers if insufficient detail was provided in the abstract to make a decision, were retrieved and read (see Figure 1). Full texts were read by one reviewer (N.D.), and a random selection of full texts excluded (20%) and all included texts were independently read and evaluated by a second reviewer (L.M.). Any disagreement or uncertainty about inclusion was assessed by a third reviewer (S.I.). A rapid appraisal of non-English language articles, using their English abstracts, was performed to ensure that any important articles were not excluded.

### Data extraction and analysis

We followed the guidance on conducting narrative synthesis from the ESRC methods programme, although not all steps were relevant to the current review,^34^ such as the development of a theoretical model. The steps included developing a preliminary synthesis and extracting relevant data from the included papers, which was completed by one reviewer (N.D.) using a standardised form and tabulation (see Table 2) and textual description in the first instance. Data extracted included author, year, country, design, participant numbers, data analysis and the main themes identified by the authors of the papers.

**Table 2.** Description of included studies.

Author	Year and country	Study design	Number/type of participants	Type of analysis	Main themes
Holley et al.^39^	2009, USA	Mixed methods – chart review, telephone/face–face interviews	13 caregivers in the face–face interview	Content analysis	Preferences about the location of care; ease of access to a geriatrics and palliative care expert; transitions of care
Gessert et al.^40^	2006, USA	Focus groups	38 family members	Thematic analysis	Attitudes towards death; attitudes towards prolonging life; drawing the line
Lawrence et al.^41^	2011, UK	In-depth interviews	27 bereaved family members, 23 care professionals	Constant comparison method	Meeting physical care needs; beyond task-focussed care; planning and communication with family
Thuné-Boyle et al.^42^	2010, UK	Semi-structured interviews	20 relatives of people with advanced dementia	Framework analysis	Illness awareness; communication; pain awareness; attitudes towards end-of-life treatments and quality of life; hospitalisation
Caron et al.^43^	2005, Canada	In-depth interviews	24 caregivers, current and bereaved	Grounded theory: constant comparison and dimensional analysis	Quality of the relationship; frequency of contact; values and beliefs; level of trust
Dening et al.^44^	2012, UK	Nominal group technique	6 people with dementia, 5 carers and 6 dyads of people with dementia and their carers	Content analysis	Good quality care; independence and control; perceptions of burden and caring
Forbes et al.^45^	2000, USA	Focus groups	28 family members of people with dementia	Content analysis	Emotional effect; insult-to-life story; two faces of death; values and goals regarding end-of-life treatments; the unrecognised trajectory of dying
Treloar et al.^46^	2009, UK	Mixed method – semi-structured questionnaire and interviews	14 carers of people with dementia	Thematic analysis	Bereavement; essential carer characteristics; required resources (professional expertise and necessary equipment); funding and financial control; feeding; medication; availability of support services; end-of-life care and place of death

Thematic analysis was used to identify and analyse the main themes of the included studies, using a coding strategy according to the principles of Corbin and Strauss.^47^ Regular debriefing and discussion of themes among three reviewers (N.D., L.M. and S.I.) and additional discussions with a fourth reviewer (G.R.) were used to enhance the reliability of the findings, until consensus about themes was reached.^35,48^ The reviewers were a multi-disciplinary team of two general practitioners (GPs), one researcher with a background in Anthropology and a researcher with a background in Psychology.

## Results

### Description of included studies

Five of the studies focussed on family carers as participants within their studies,^39,40,43,45,46^ one used both family carers and people with dementia as participants^44^ and finally, two of the studies interviewed both family carers and professionals.^41,42^

The studies spanned a variety of settings with two focussing on the person with dementia in their own home.^39,46^ Both of these studies were evaluations using interviews with carers about programmes designed to encourage care at home for people with dementia at the end of their life: the Palliative Access through Care at Home (PATCH) programme^39^ based in America and work undertaken by psychiatrists in the United Kingdom.^46^

Two of the studies focus on decisions which are made by carers who have relatives in nursing homes.^40,45^ Only one study examined care received in hospitals, which was conducted to inform an intervention to improve hospital care for dementia in the United Kingdom.^42^ A further study explored relationships between health-care providers and carers in long-term care facilities in the United States,^43^ as part of a much larger study on decision-making in carers of people with dementia.^49^

The remaining two studies are spread across different settings; Dening et al.^44^ recruited from memory clinics which would see patients from both community (own home) and care homes. They focussed on exploring persons with dementia and carers’ preferences about EOLC. Lawrence et al.^41^ recruited participants from a range of settings – hospitals, care homes and continuing care units – and examined the definition of good EOLC for dementia across these settings.

### Quality appraisal

For the review, the literature was appraised for quality using the Critical Appraisal Skills Programme (CASP) tool.^50^ No studies from the review were excluded based on the results of their quality appraisal. However, using CASP did help to show the range of quality among the studies. Some of the studies did not describe the methodology of the study very well,^46^ and some fail to explain why they have chosen certain methods, for example, focus groups as opposed to interviews,^40^ which have both advantages and disadvantages in EOLC research.^51^ The lack of detail and clarity of some may have been due to the restrictions on word limits when writing for publication.^52^ Similar criticism also apply to the data analysis sections with two studies not stating the type of analysis used,^40,46^ but based on the detailed description of the analysis by both sets of authors, the reader can assume that they both used a method of thematic analysis.

The ‘Results’ sections of the papers were predominantly well discussed and supported by interesting and relevant quotes. However, two of the articles had a mixture of professionals and family carers’ views,^41,42^ which sometimes made it difficult to understand whether the discussion was directly applicable to carers or based on carers’ views. Some studies provided themes with a discussion, supported by very few quotes^39,41,45^ or quotes which did not address the entirety of the topic being discussed.^39^

### Results of the synthesis

Two themes were identified from the thematic analysis as common to all papers included in this review: a family’s varying degree of acceptance that their relative with dementia was actively dying and a family’s idea of the appropriate level and purpose of treatment for their relative. These two themes are heavily related and together construct an overarching theme ‘a family’s belief of death and their choice of treatment’.

#### A family’s belief of death and their choice of treatment

The papers demonstrated variation among family carers about what they perceived to be high-quality EOLC for dementia, with different preferences about the level of treatment which should be provided, either more active and invasive treatments such as artificial nutrition and hydration or more palliative approaches aimed at achieving comfort.

The first theme consisted of three constructs about the acceptance of death, situated along a spectrum from acceptance to denial, with carers who were ambivalent/unsure about their attitude towards the death of their relative positioned between. It should be emphasised that these are loose constructs and are not intended to be a simple categorisation of carers in a reductionist form. Many carers may neither accept nor deny that their relative is actively dying, and to suggest such would be an oversimplification of the emotions and beliefs of carers, with carers potentially oscillating between different positions of acceptance.

The second theme appeared to be discussing similar constructs related to acceptance of their relative as actively dying, but reflected a preferences for treatment, therefore what the reader may infer as the carer’s opinion about high-quality care. Again, views spanned a spectrum from no or minimal treatment, aimed only at symptom modification or relief, to active treatment, aimed at ‘cure’.

Gessert et al.^40^ explored the attitudes of families towards the death of their relative with dementia and EOLC, focussing on differences between rural and urban families. Many of the rural respondents clearly accepted death and it was natural for their relative to die, consequently preferences of treatment were for rather minimal and non-invasive treatments:

Most rural focus group participants voiced unqualified acceptance of death. Death was characterized as natural, often with references to ‘ going to sleep’, or using language that described death as the accepted and expected ‘ next step’ in the life of the elder. (p. 3)^40^

Their urban counterparts, however, were less accepting of death, and some even denied death to some extent, opting for treatment which was more active or curative:

Several urban respondents rejected hospice and palliative care options that had been offered to them and regarded hospice as inappropriate under their specific circumstances because hospice care was seen as ‘not aggressive enough’. ‘The hospice lady cornered my one brother … and she really tried to get him to sign on the line … we can’t go along with that thinking … they had the hospice person come every day and I mean I’m fully aware of what hospice is …’. (p. 5)^40^

The variation of acceptance and denial of death and dying was discussed in many of the studies, represented by families’ preference of treatment for their relative.^40–46^ Some carers discussed various types of treatment that could be delivered to a person with dementia at the end of life, including antibiotics, artificial nutrition and hydration through feeding tubes, mechanical ventilation and resuscitation. Some were adamant that they did not want any treatment for their actively dying relative:

At one point, they thought that maybe she had pneumonia. So, I met with the doctor. She said, ‘We can treat it, we can give her antibiotics and that will pro-long her life’. I said no because I knew that it meant that she would suffer longer and because of how she was, there was no point in prolonging. (p. 240)^43^

Some believed that there were treatment options acceptable at the end of life, in particular those seen as less invasive and used to maintain comfort, such as the use of antibiotics for an incident of pneumonia:

[…] treatments such as antibiotics and feeding tubes should be offered as long as patients were comfortable. This was often the case even for those relatives who were against resuscitation. (p. 273)^42^

However, others appeared to perceive antibiotics as a life-sustaining treatment compared to those who considered it a measure to simply enhance comfort:

Death was forbidden if the resident had any treatable conditions, such as a urinary tract infection or pneumonia, or if a physician had not declared the resident as terminal. (p. 254)^45^

With comfort and treatment often causing internal conflict and turmoil for many families, reflecting an uncertainty about not only the acceptance of death and dying but also their preference for treatment:

If she got pneumonia next week, I’m not sure of what I would do. I would probably still have her treated. (p. 4)^40^

There were also those who favoured more intensive and potentially invasive treatments which are aimed at cure, reflecting those who are perhaps in denial about their relative actively dying:

I think I would want the feeding tube because the rest of her body wasn’t going … that was the only thing that was holding her up was the fact that she wasn’t eating. Then she’s starving herself to death rather than dying, you know … I would ask for a feeding tube until her body seemed to be all complete, be going, you know. I don’t want her to starve. I think that would be more painful in a way, you know. More harder. (p. 256)^45^

With some thinking that their relatives deserved more in terms of treatment and not simply be left to die because they were old:

The carers felt that medical decision-making and the use of end-of-life care pathways could invalidate their ACPs:

[…] you are put on the short count to death row [End of Life Care Pathways] … I think a lot of elderly people are put on that path because it happens to be convenient … just because they are old basically, the plug is pulled … that decision can sometimes be made too early. (p. 413)^44^

Urban families from Gessert et al.,^40^ who were more likely not to accept their relative was actively dying, suggested that measures such as ventilation could be used but only on a short-term basis, suggesting more of a position of acceptance and a preference for comfort:

Urban family members expressed a range of attitudes toward feeding tubes and respirators but were receptive to their use under defined conditions, as long as they did not become permanent. ‘Q[interviewer]: You would try to offer her healing with what? A[respondent]: Well, I mean the medication. I mean whatever antibiotics. I mean as if she were anyone else. Q[interviewer]: As if she were you? A[respondent]: Yes. Q[interviewer]: Complete with hospitalization and using the breathing machine. The ventilator? A[respondent]: Even using the breathing machine if it’s not for … if you don’t foresee long-term use of … that machine’. (p. 5)

Finally, a group appeared to want comfort measures and treatment that were not aimed at cure but more symptom modification; however, they struggled with describing this in detail:

Participants discussed the desire for preserving dignity, promoting comfort, and ensuring good day-to-day care, but had difficulty incorporating goals such as comfort care into specific treatment plans. Comfort was a dominant goal and emerged in discussions of pain and suffering. Examples participants gave were, ‘I don’t want my mom to suffer’. ‘I hope she can go peacefully in her sleep and not have to suffer’. Family members were unable to move beyond a broad description of comfort to specific care options. (p. 255)^45^

Why do family carers have these opinions of care and stance of acceptance of the dying process?

#### The relationship with professionals as a core component of care quality

Across the papers, the authors appeared to suggest that the relationship carers had with professionals was seen as important. The relationship with professionals was important in many aspects, including contact, the provision of information and support/relief/reassurance for the carers. Professionals varied among the papers and included GPs, social workers, district nurses and other types of physicians.

Carers felt that an important aspect of a professionals’ role was simply contact between them and the family and the person with dementia:

One of the greatest dissatisfactions expressed by the family caregivers who participated in this study relates to the limited contact between themselves and the providers working with their family member. (p. 238)^43^

Some families were particularly happy when professionals spent the time to get to know the person with dementia and what they are like, subsequently building an element of trust:

There was a general fear and uncertainty with a lack of trust in medical decision-making:

… being sure that treatment is in my best interests … It means that you have got to trust in people who make the decision … (p. 413)^44^

Carers alluded that contact did not simply mean getting to know the person with dementia but also having formal meetings with them as carers:

[…] need, perceived by carers, for someone who would visit regularly, advise and bring in other people. (p. 342)^46^

Others did not feel the need for frequent but rather more regular contact such as once a year:

Certain people hoped for fairly frequent meetings (frequent need to validate perceptions and receive answers to certain questions), whereas for other people, a regular, but not necessarily frequent, contact (i.e., once a year) would have been sufficient. (p. 239)^43^

The purpose of these meetings or interaction with professionals varied. For some, it was provision of information about the current condition of their relative or simply information about dementia and the kind of care now appropriate that they were seeking, allowing them to make decisions about treatment and preparing them for the death of their relative:

Well, for me, I think that in terms of the relationship [with the] family, it might have been good to have meetings with the staff, to see what is going on with [my relative], treatments, the evolution of the disease as well as getting to know each other a little bit. (p. 238)^43^

If families were not provided with information about their relatives’ condition, dementia, what palliative care entails and what to expect at the end of life, carers may lack vital knowledge and understanding:

Families were aware that their relative’s memory problems would deteriorate in the future but were often unaware of the terminal nature of dementia and its physical consequences. (p. 266)^42^

However, these meetings were also seen as a way of providing vital support and ‘relief’ for the carers, with professionals answering any concerns:

In summary, caregivers appreciated the easy access to a knowledgeable practitioner that PATCH provided. Having an expert in geriatrics and palliative medicine physically present in the home provided reassurance to patients and caregivers. (p.1929)^39^

To provide information and reassurance, professionals would need to be knowledgeable and respected, which sometimes was not the case:

For many, this typified hospital staff’s lack of understanding of the needs of people with dementia and what to do to meet them:

‘There was no people feeding them and I went, I used to go in and feed her and they said, “Oh no, she’s here to be rehabilitated, you shouldn’t feed her, it’s spoiling her, she can do it herself,” well … she couldn’t do it at all and as I say her eating was getting worse and worse at that stage.’ (p. 418)^41^

Many relatives also said that hospital staff did not seem to understand the needs of their relative: It was astonishing how little understanding the [hospital] staff had of him, of his condition, even though I did tell them … but I wasn’t there all the time … so they were asking him questions, but obviously, he didn’t even try to answer. So they were asking questions and then gave up and left him … and they seem to be nervous of him. (p. 267)^42^

But there was an indication among the papers that this was more than just the knowledge of hospital staff about feeding and understanding the needs of the patient and their family, but comprehensive knowledge of dementia, palliative care and EOLC:

A parallel concern was having access to a practitioner trained in geriatrics, palliation, and end-of-life care. One caregiver said: The only thing is that what we really needed was an expert geriatrician who knows a lot about the medicines. (p. 1929)^39^

Conflict between professionals and families also appeared to cause difficulties with treatment choice and the acceptance of death. This may suggest that although some may be influenced and helped by the information provided by professionals, some will already have made up their minds and other factors must therefore influence their ideas about quality EOLC:

In fact, several of the rural respondents anticipated that they might have to fight with medical personnel to prevent interventions in the dying process:

‘If that ever happened … I’d have to fight the doctors and everything else because they wouldn’t go for it. … Yeah, the medical side would … want her to be treated’. (p. 4)^40^

#### Emotional and commitment pressures of caring

The papers briefly described some of the emotional strains and commitment pressure which can harbour carers when caring for a relative with dementia, despite this not being a focus of the review, a link was identified between emotional strains and commitment pressures and carers’ choice of treatment and acceptance of death.

Adult children find caring in particular demanding, striking a balance between ‘normal’ family life with their children and with caring for their relative. Often unable to devote as much attention as needed:

Family members expressed the pain of splitting themselves between their own immediate family, their personal needs, and the needs of the family member in the nursing home. A daughter said, ‘I mean it’s totally on my shoulders too. And sometimes the amount of guilt is there, I mean it just weighs me down so much. But then I have to split myself, I still have kids at home, and I have to split myself there too’. (p. 254)^45^

Many family carers also experience strains of guilt, with some feeling torn between their thoughts of acceptance of death, which could lead them to be seen as an ‘agent of death’, and the tragedy of death, which Forbes et al.^45^ describe as the two faces of death, creating a sense of uncertainty about death and treatment preference:

Torn between death as an acceptable blessing versus a forbidden tragedy, family members did not want to be an ‘agent of death’. Family members wanted peace of mind, to believe that they had done everything possible and that death was beyond their control (p. 255)

But their feelings of guilt were not restricted to guilt towards the person with dementia but an element of feeling guilty about their preference of care and being judged by the professionals, with some fearing a lack of support from the system as a whole:

I was afraid of being judged at times. (p. 240)^43^

#### Family carers’ ability to think about death and dying

Some papers suggest that family carers may struggle to discuss EOLC and the prospect of their relative dying having previously not discussed or thought about what may happen in the future:

[…] but I haven’t broached the subject of the future. I just can’t bear to think about it really. (p. 268)^42^

Some were unsure about what to do for the best:

So does the oxygen just make them feel more comfortable? What is the purpose of the oxygen? I mean, I wouldn’t know, I would have to ask a lot of questions, I couldn’t make that decision. (p. 5)^40^

However, for some it was less of a fear, but uncertainty about the future and how to plan for it. They were concerned that the plans would not remain the same with the doctor possibly changing care plans or if the person with dementia would have changed their mind had they been able to:

If he made it clear that he didn’t want to be resuscitated, whether he’s changed his mind about that now … people do sometimes, you know. I thought he would change his mind about that, but he was quite adamant at the time that he didn’t want to be (p. 272)^42^

There appeared to be some, perhaps those more accepting towards death who did not struggle to think and plan ahead:

One family member commented that advance care planning might alleviate the burden associated with this role. ‘I think it makes it easier for the carer if they know because then you haven’t got that moral dilemma. Because like I was placed in … was I stopping her having her last chance of life by not letting her go to [hospital] for the dehydration? … Would she have wanted it? You know you tear yourself in pieces’. (p. 420)^41^

## Discussion

The small number of papers which were eligible for this review demonstrates clearly the lack of research attention to family carers’ experiences of EOLC for people with dementia. Contrary to previous reviews of EOLC and dementia, which found an abundance of work from North America, the current review found an even split between studies from North America and the United Kingdom.^28,53^ This is especially encouraging as the World Health Organization (WHO)^54^ stated that the ‘transferability of learning and experience is contextual’; it is not necessarily true that the success of a quality improvement in one setting/country can be the same in another.

### A family’s belief of death and their choice of treatment

The literature does not provide enough evidence to define high-quality EOLC for dementia from a carer’s perspective. The studies included in this review show that carers’ attitudes and beliefs vary greatly. For example, there is a range of acceptance of death of the person with dementia, from complete acceptance, and in some cases even a wish for death, through to complete denial of death. Carers’ views appeared to be aligned upon a spectrum, perhaps moving along this spectrum through the course of the ‘caring career’. Many carers often did not know what to do for the best, how they feel or how the person with dementia would feel and can be left feeling ambivalent and often trapped between two extremes, as previously suggested by Peacock^28^ in her review. Peacock notes that there may be other studies which describe this but simply do not name feelings as ambivalence. The current review supports this and has reflected this in the spectrum of acceptance of death. However, this review has found that this ambivalence is not only about acceptance of death but also about the level of appropriate treatment for EOLC.

### The relationship with professionals as a core component of care quality

A range of different professionals are required to help a person with dementia receive EOLC that is holistic in its approach.^55^ This review highlights the importance of communication among the triad of carer, person with dementia and professionals. We agree with Hennings et al.^26^ that communication often does not happen at times of crisis, leading to rushed decisions and potentially poor quality care. However, as our review highlights, it is not enough for professionals to just communicate, they must also be knowledgeable and respected. This is often difficult as many professionals have little training and opportunities to train in both dementia and palliative care, a shortcoming which is often made worse by work time constraints,^56,57^ with many wanting to know more.^58^ However, as Davies et al.^58^ suggest training is not simply acquiring new technical skills, it is also about confidence and personal skills, with many professionals having difficulty supporting carers.^59^

### Emotion and commitment pressures of caring

Caring can be a demanding and difficult role which many have had forced upon them.^60^ As shown in the ‘Results’ section of this review, caring for someone who is actively dying and who is unable to communicate for themselves as in dementia can cause feelings of guilt, not simply due to the decisions about treatment they have to make but also because of their inability to spend time with their family, for example, in cases of adult children. But it is not simply relationships with other family members which may become strained. The commitment needed for caring can affect the carer’s social life, leaving feelings of being socially isolated.^61^ Some have suggested that the closer an individual is to the person they are caring for, the more difficult it can be psychologically and emotionally.^62^

### Family carers’ ability to think about death and dying

Our review found that many carers were not able to think about the death and dying process of their relative. This corresponds to perceptions that professionals have about families’ reluctance to discuss planning for end of life,^63^ but also reluctance among professionals themselves to discuss EOLC.^64^ Similarly, Sampson et al.^65^ designed an intervention to improve EOLC through advance care planning (ACP), but they struggled to engage carers in ACP, with only 7 carers making ACPs out of a possible 22. More research is needed to understand the thought processes and emotions of carers of people with dementia, and we agree with Dening et al.^66^ that more understanding is needed about how to better engage people with ACP. But also acknowledge that ACP may not be for everyone and therefore approaching ACP should be carefully considered by professionals before engaging with families and people with dementia.

EOLC for people with dementia requires input from many different professionals, social workers, GPs, district nurses and potentially specialists such as palliative medicine specialists and geriatricians. This team and their interaction are important, providing vital and often valued information to the family carers. A families’ ability to think about death and dying may be facilitated if families have a key member in order to liaise with or contact, as part of Forbes et al.’s^45^ results suggest a consistent provider would be helpful. This exemplifies once more the importance professionals play for some carers.

### Limitations

Despite a systematic approach taken to search for papers in this review, there may have been papers which were missed. Furthermore, the studies which were found had differing methodologies, with authors from a range of backgrounds professionally making them difficult to synthesise.

This is a secondary analysis using thematic analysis methods on data which have been removed from the context of the carers’ original interviews in the studies. The data extracted for this review were both original quotations from the carers in the original research in addition to the text and discussion around this from the authors of the original studies. Therefore, some of the interpretation and analysis within this current review is based on previous authors’ interpretations, making it a step removed at times from family carers. Finally, this review is limited by a general paucity of literacy evidence from which to make conclusions.

### Implications for research and practice

The results from the included studies of this review generate a rather two-dimensional picture of carers’ experiences, and more information from carers is needed to provide greater in-depth details of experiences.

This review demonstrates variations in individual’s preferences of care and their acceptance or denial of death, which may relate to their attitudes towards high-quality EOLC. We have alluded to the differences which may exist between spousal carers and adult children, due to differing priorities, such as an adult child’s young family. Although previous research has identified differences in the grieving process, strain and satisfaction with support services,^17,18,67,68^ we know relatively little about how their views on quality of care and preferences of care may differ. It would also be valuable to understand differences between those who are currently caring compared to those who are bereaved, which has not yet been explored.

With many carers not accepting the death of their relative, this creates concern about their well-being and reaction to the death. Hudson^59^ has urged for greater investment in bereavement research for carers, with bereavement potentially starting before death and during EOLC,^69^ and our study strengthens this call. Quantitative research by Kiely et al.^16^ has shown that bereavement may be no more important in dementia than in other diseases but grieving processes may be longer. Quantitative studies such as this support the need for more qualitative research to understand the complex issue of bereavement and grief.^16^

The recent withdrawal of the LCP has left a gap in the field of guidance and frameworks in the United Kingdom for delivering EOLC for all conditions not just dementia. However, recommendations from the independent review of the LCP include developing disease-specific guidelines,^32^ and we would agree that specific guidelines and frameworks are required. Encouragingly, this comes at a time when the European Association for Palliative Care (EAPC) has developed a set of recommendations on palliative care for people with dementia.^70^ With family carers potentially responsible for the failure of the LCP, it is vital to capture and incorporate the variation in carers’ views on what is high-quality EOLC when developing new guidelines and frameworks. Those developing such guidelines and frameworks need to ask themselves ‘have we understood the variation in views?’. However, more work is needed to gain a better understanding of what carers need, want and believe to be high-quality EOLC across all settings of care for people with dementia.

## Conclusion

This review is important in highlighting that there is no simple categorisation of carers who are accepting or denying the death/dying of their relative with dementia. This study demonstrates that carers are independent in their thought processes and opinions. This must be respected by all manner of professionals involved in the EOLC of not only an individual with dementia but a family when devising and applying a care plan which needs to be personalised to that individual and family.
